# The Ramachandran plots of glycine and pre-proline

**DOI:** 10.1186/1472-6807-5-14

**Published:** 2005-08-16

**Authors:** Bosco K Ho, Robert Brasseur

**Affiliations:** 1 Department of Pharmaceutical Chemistry, University of California San Francisco, 600 16th St, San Francisco, CA 94107, USA; 2 Centre de Biophysique Moléculaire Numérique, 2 Passage des déportés, B-5030 Gembloux, Belgium

## Abstract

**Background:**

The Ramachandran plot is a fundamental tool in the analysis of protein structures. Of the 4 basic types of Ramachandran plots, the interactions that determine the generic and proline Ramachandran plots are well understood. The interactions of the glycine and pre-proline Ramachandran plots are not.

**Results:**

In glycine, the ψ angle is typically clustered at ψ = 180° and ψ = 0°. We show that these clusters correspond to conformations where either the N_i+1 _or O atom is sandwiched between the two H^α^ atoms of glycine. We show that the shape of the 5 distinct regions of density (the α, α_L_, β_S_, β_P _and β_PR _regions) can be reproduced with electrostatic dipole-dipole interactions. In pre-proline, we analyse the origin of the ζ region of the Ramachandran plot, a region unique to pre-proline. We show that it is stabilized by a CO_i-1_···C^δ^H^δ^_i+1 _weak hydrogen bond. This is analogous to the CO_i-1_···NH_i+1 _hydrogen bond that stabilizes the γ region in the generic Ramachandran plot.

**Conclusion:**

We have identified the specific interactions that affect the backbone of glycine and pre-proline. Knowledge of these interactions will improve current force-fields, and help understand structural motifs containing these residues.

## Background

The Ramachandran plot [1] is the 2d plot of the φ-ψ torsion angles of the protein backbone. It provides a simple view of the conformation of a protein. The φ-ψ angles cluster into distinct regions in the Ramachandran plot where each region corresponds to a particular secondary structure. There are four basic types of Ramachandran plots, depending on the stereo-chemistry of the amino acid: generic (which refers to the 18 non-glycine non-proline amino acids), glycine, proline, and pre-proline (which refers to residues preceding a proline [2]). The generic and proline Ramachandran plots are now well understood [3] but the glycine and pre-proline Ramachandran plots are not.

The generic Ramachandran plot was first explained by Ramachandran and co-workers in terms of steric clashes [1]. This has become the standard explanation for the observed regions in the Ramachandran plot [4,5]. However, recent studies found significant discrepancies between the classic steric map and the Ramachandran plot of high-resolution protein structures [6-9]. These discrepancies have now been resolved. The first discrepancy is that the N···H_i+1 _and O_i-1_···C steric clashes in the classic steric map have no effect in the observed Ramachandran plot [3]. By removing these steric clashes, a better steric map can be constructed. The second discrepancy is that the Ramachandran plot cluster into distinct regions within the sterically-allowed regions of the Ramachandran plot [8,10]. These clusters have now been explained in terms of backbone dipole-dipole interactions [3,11,12].

The proline Ramachandran plot has been reproduced in a calculation [13]. The proline Ramachandran plot is severely restricted by the pyrrolidine ring, where the flexibility in the pyrrolidine ring couples to the backbone [14].

**Figure 1:**
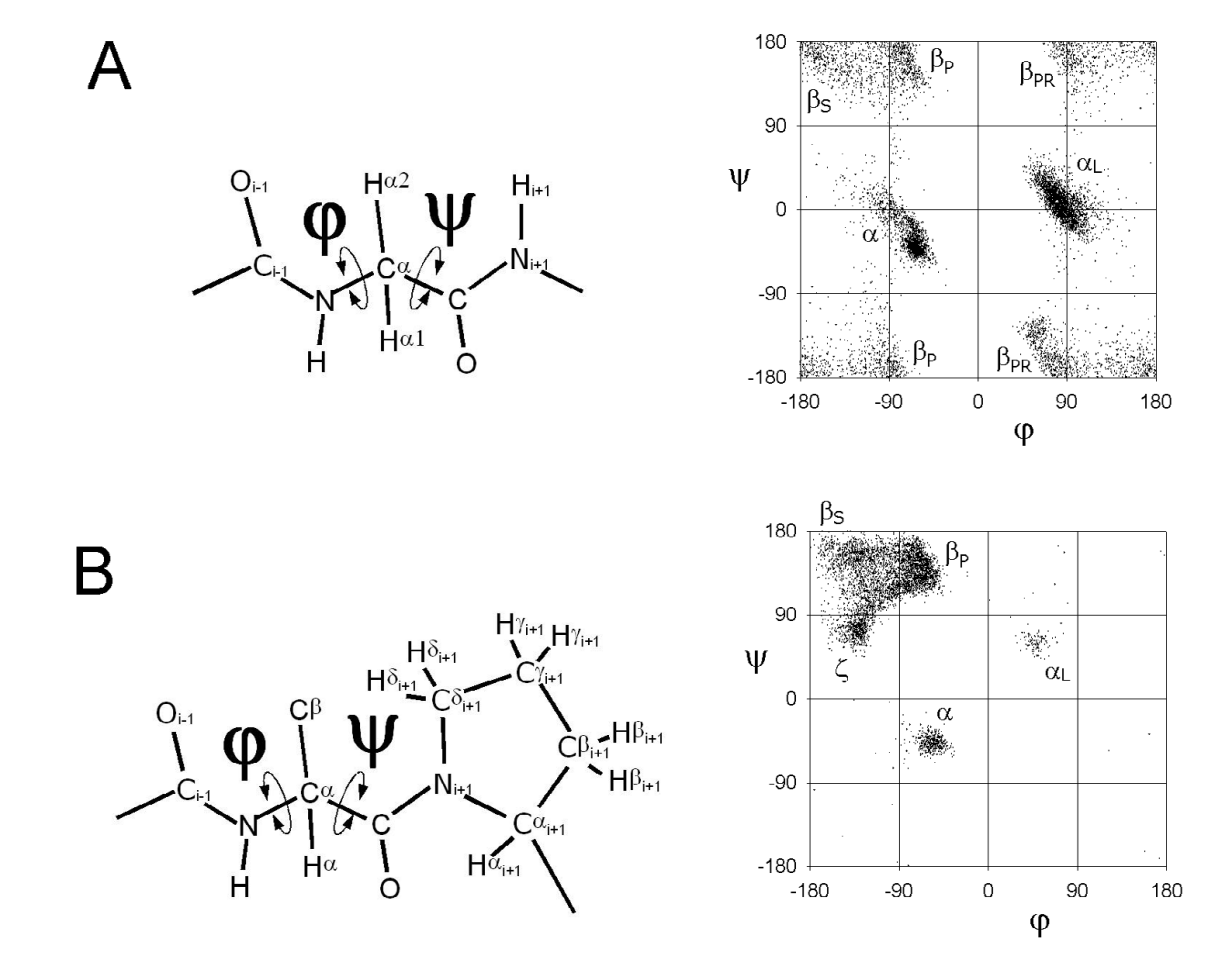
**Backbone conformations of glycine and pre-proline. **Backbone schematic (left) and observed Ramachandran plot (right) of (A) glycine and (B) pre-proline. Taken from the data-set of Lovell et al. (2003). The clustered regions are labeled on the Ramachandran plots.

**Figure 2:**
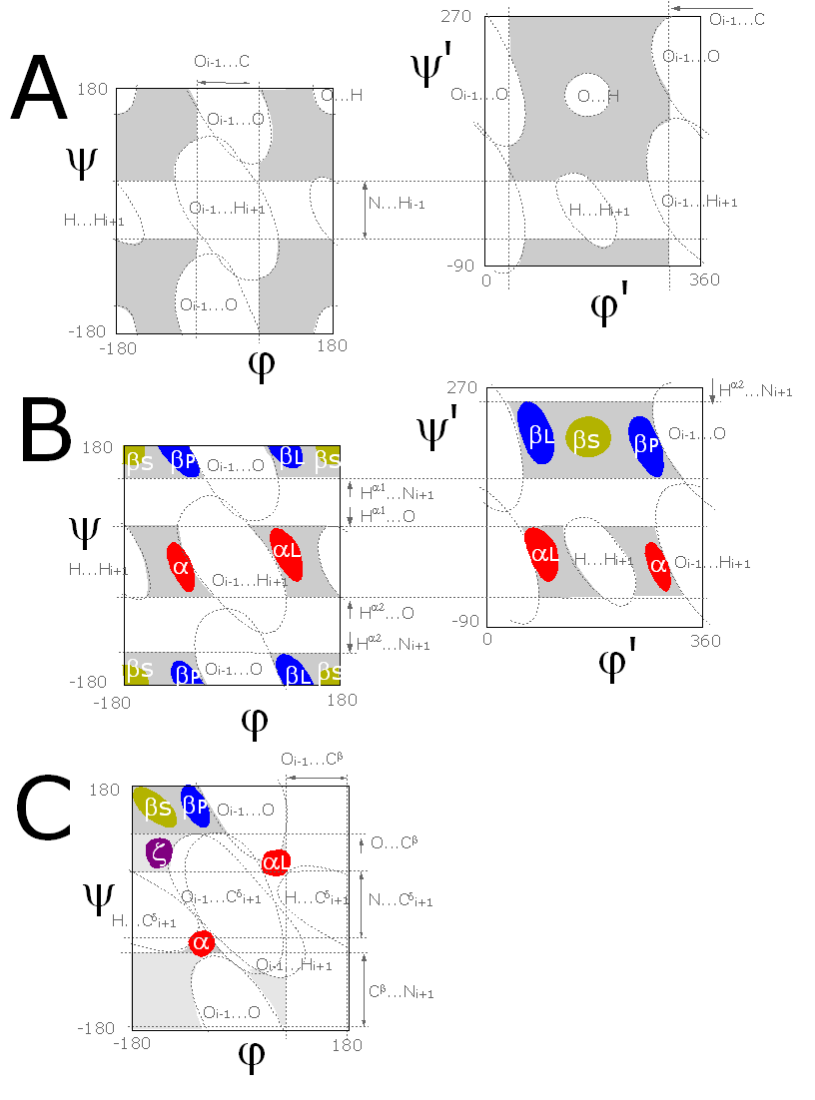
**Schematic of the Ramachandran plot. **(A) original steric map of glycine, in standard (left) and shifted (right) coordinates; (B) revised schematic of glycine, in standard (left) and shifted (right) coordinates; (C) pre-proline. The clustered regions are: grey – sterically allowed; red – α and α_L_; yellow – β_S_; blue – β_P _and β_PR_; purple – ζ. See text for explanation of the regions.

The observed glycine Ramachandran plot has a distinctive distribution (Figure 1A) quite different to the generic Ramachandran plot. An early attempt to explain the observed Ramachandran plot in terms of a steric map of glycine [15] (Figure 2A) fails to account for the observed distribution. It does not explain the observed clustering at ψ = 180° and ψ = 0°, nor the clustering into 5 distinct regions [8]. Using a molecular-dynamics simulation of Ace-Gly-Nme [16], Hu and co-workers found that the glycine Ramachandran plot generated by standard force-fields reproduced the original steric map but not the observed Ramachandran plot. They calculated a somewhat better result with a quantum-mechanics/molecular-mechanics model, which reproduced the observed clustering along ψ, but not the partitioning into the 5 clusters. In this study, we identify the specific interactions that define the observed glycine Ramachandran plot by studying the conformations of glycine in the structural database. We test these interactions with a simple model based on electrostatics and Lennard-Jones potentials.

Although the overall shape of the pre-proline Ramachandran plot (Figure 1B) is well understood, there exists a region unique to pre-proline that remains unexplained. The basic shape of pre-proline was predicted by Flory using steric interactions [17]. This was later confirmed in a statistical analysis of the protein database [2]. However, the statistical analysis also revealed the existence of a little leg of density poking out below the β-region (Figure 1B; purple in Figure 2C), which Karplus called the ζ region [10]. More recent calculations using standard molecular mechanics force-fields reproduced the energy surface of the original Flory calculation [13,18] but not the ζ region. In this study, we focus on the physical origin of the ζ region.

## Results

### A non-redundant PDB data-set

To extract the statistical distributions of the glycine and pre-proline Ramachandran plots, we chose a high-resolution subset of the PDB [19] provided by the Richardson lab [9] of 500 non-homologous proteins. These proteins have a resolution of better than 1.8 Å where all hydrogen atoms have been projected from the backbone and optimized in terms of packing. Following the Richardsons, we only consider atoms that have a B-factor of less than 30.

### Regions in the glycine Ramachandran plot

Glycine is fundamentally different to the other amino acids in that it lacks a sidechain. In particular, glycine does not have the C^β ^atom, which induces many steric clashes in the generic Ramachandran plot. We call the hydrogen atom that is shared with the other amino acids, the H^α1 ^atom. We call the hydrogen atom that replaces the C^β ^atom, the H^α2 ^atom. The absence of the C^β ^atom allows the glycine Ramachandran plot to run over the borders at -180° and 180° (Figure 1A).

**Figure 3:**
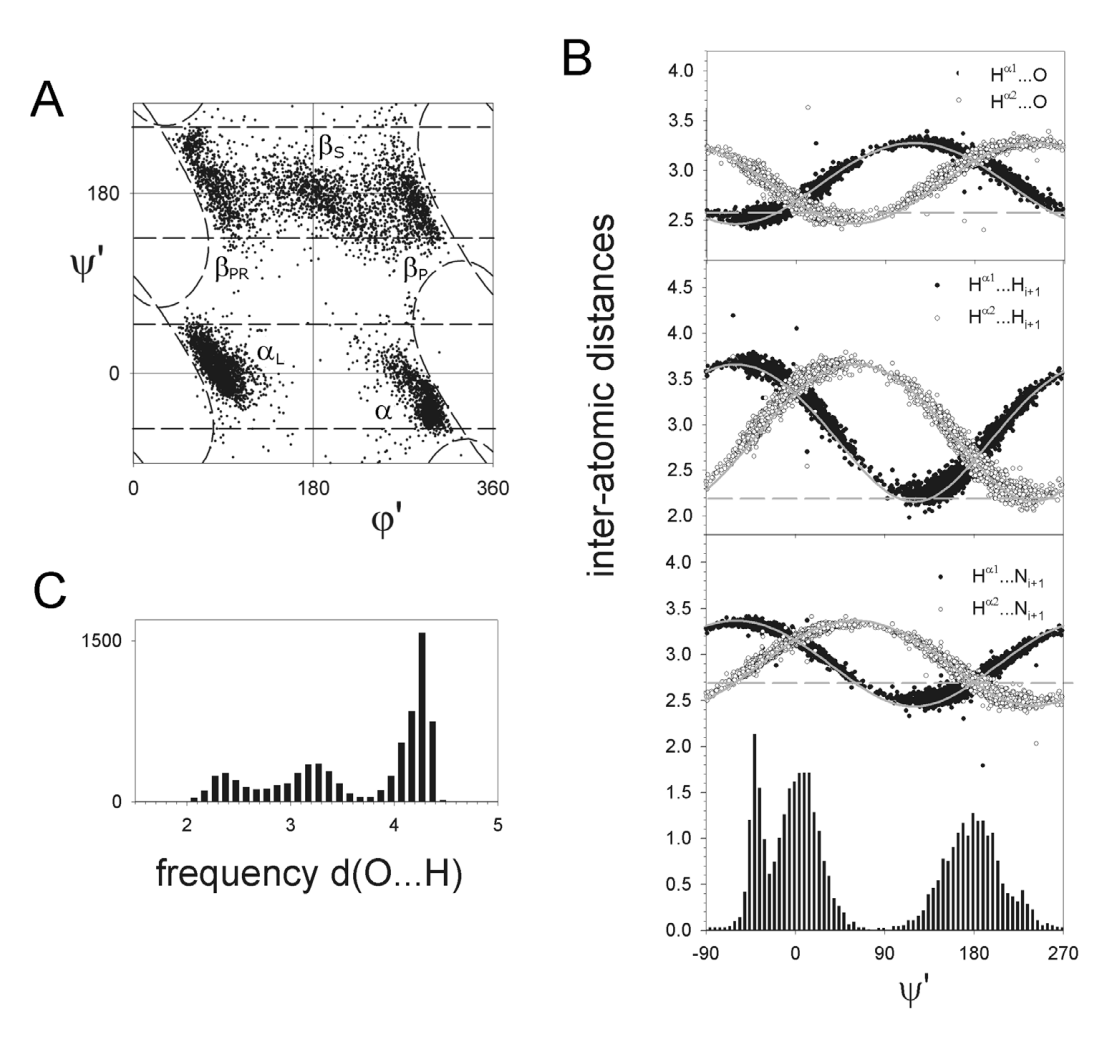
**Glycine parameters. **(A) The Ramachandran plot in shifted coordinates φ'-ψ'. The dashed lines show the steric clashes that define the boundaries of the observed densities (Figure 2B describes the specific interactions). (B) The distributions of various inter-atomic interactions as a function of ψ'. The dashed line show the limit of the VDW diameters. The grey line gives the model curve calculated with ideal geometry. At the bottom is the frequency distribution of the ψ' angle. (C) Frequency distribution of the inter-atomic distance d(O···H). There are 3 peaks, of which, the smallest at d(O···H) = 2.4 Å, which corresponds to the β_S _region.

The observed glycine map has 5 regions of density [8]. In order to display the observed density in one continuous region, we shift the coordinates from φ-ψ to φ'-ψ' where φ': 0° < φ' < 360°, and ψ': -90° < ψ' < 270°. With the shifted glycine Ramachandran plot (Figure 3A), we can clearly identify the different regions. Along the horizontal strip ψ' ~ 180°, there are three separate regions. One of these is an elongated version of the β_P _region of the generic Ramachandran plot. The β_P _region corresponds to the polyproline II structure, which forms an extended left-handed helix along the protein chain [20]. The β_PR _region is a reflection of the β_P _region where a sequence of glycine residues in the β_PR _conformation will form a right-handed helix. Finally, there is a region that corresponds to the β_S _region of the generic Ramachandran plot. This region corresponds to the extended conformation of residues in β-sheets. However, the glycine β_S _region, centered on (φ', ψ') = (180°, 180°), is slightly displaced from the β_S _region of the generic Ramachandran plot. There is also the diagonal α and α_L _regions (Figure 3A), which are associated with helices and turns [3]. Unlike the generic Ramachandran plot, the glycine α region is symmetric to the α_L _region [8,21]. In the generic Ramachandran plot, there is also a γ region corresponding to the hydrogen bonded γ-turn [12]. The glycine Ramachandran plot does not have any density in the γ region.

### Steric interactions in glycine

The original steric map of glycine (Figure 2A) [15] fails to explain large parts of the observed glycine Ramachandran plot (Figure 1A). In the observed glycine Ramachandran (Figure 3A), there are two large excluded horizontal strips at 50° < ψ' < 120° and -120° < ψ' < -50°, which are not excluded in the glycine steric map (Figure 2A). Conversely, the glycine steric map excludes a horizontal strip at -30° < ψ' < 30° (Figure 2A), but this region is populated in the observed plot (Figure 1A). There are also diagonal steric boundaries in the observed glycine Ramachandran plot (Figure 1A), whereas the steric map predicts vertical boundaries (Figure 2A).

We carried out a re-evaluation of the steric map of glycine (Figure 2B) by following the methodology of Ho and co-workers [3]. For each interaction in the glycine backbone, we consider the variation of the inter-atomic distance with respect to the φ'-ψ' angles. We compare the observed variation to the variation generated from a model that uses canonical backbone geometry. We divide these interactions into 3 categories: the φ' dependent, ψ' dependent and φ'-ψ' co-dependent distances.

For some of the interactions, the results for glycine are identical to that of the generic Ramachandran plot [3]. For brevity, we omit the analysis of these interactions and summarize the results. The excluded horizontal strip -30° < ψ' < 30°, due to the N···H_i+1 _steric interaction in the glycine steric map (Figure 2A), does not exist in the observed distribution (Figure 1A). Similarly, the O_i-1_···C steric clash in the original glycine steric map, which excludes a vertical strip centered on φ' = 0° (Figure 2A), does not exist in the observed distribution (Figure 1A). We ignore the effect of the N···H_i+1 _and O_i-1_···C steric clashes. The diagonal boundaries of the observed distribution are defined by the φ'-ψ' co-dependent steric interactions O_i-1_···O and O_i-1_···N_i+1_. In Figure 3A, we show the fit of these steric interactions to the data.

Here, we analyze the most distinctive feature of the glycine Ramachandran plot – the tendency for ψ' to cluster near 180° and 0°. We focus on the ψ'-dependent interactions. For each interaction, we first calculate the model curve of the corresponding inter-atomic distance as a function of ψ' (see Methods). We then compare the observed ψ' distribution (bottom of Figure 3B) to the curve. If a hard-sphere repulsion restricts ψ', then, in regions of ψ' where the model curve is below the van der Waals (VDW) diameter (horizontal dashed line in Figure 3B), the ψ' frequency distribution should drop correspondingly.

In the region (60° < ψ' < 100°), we find that the drop-off in the ψ frequency distribution (bottom of Figure 3B) corresponds to values of H^α1^···N_i+1 _(bottom of Figure 3B) and H^α2^···O (top of Figure 3B) that are smaller than their VDW diameters. In the region (-90° < ψ' < -60; 210° < ψ' < 270°), the drop-off in the ψ frequency distribution corresponds to regions where H^α2^···N_i+1 _and H^α1^···O are found below their VDW radii. In contrast, the values of H^α1^···H_i+1 _and H^α2^···H_i+1 _are never found significantly below their VDW diameter (middle of Figure 3B).

**Figure 4:**
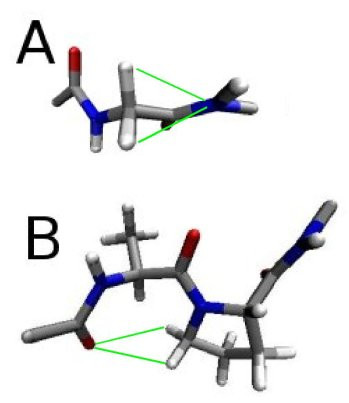
**Stick figure representation of glycine and pre-proline. **(A) glycine in the ψ ~ 180° conformation where the N_i+1 _atom is sandwiched between the two H^α ^atoms, and (B) pre-proline in the ζ conformation where the O_i-1 _atom interacts with the H^δ ^atoms of the succeeding proline.

The observed ψ' dependence in glycine is due to the H^α1^···O, H^α2^···O, H^α1^···N_i+1 _and H^α2^···N_i+1 _steric clashes. A simple interpretation is that the ψ' dependence in glycine arise from conformations that place either the N_i+1 _or O atom between the two H^α ^atoms (Figure 4A). The observed limits in the distributions have been drawn in Figure 3A as horizontal lines.

**Figure 5:**
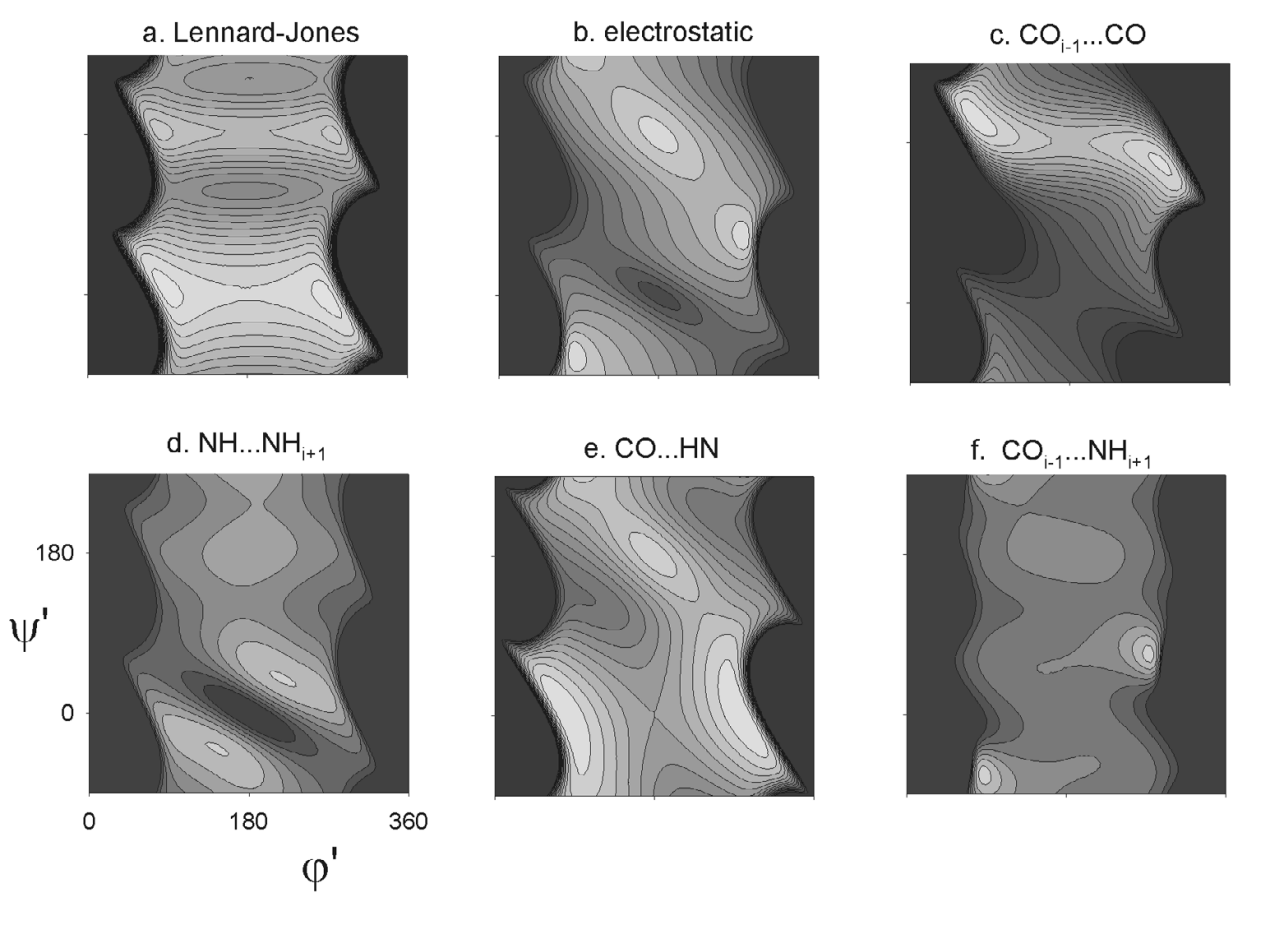
**Dipole-dipole interactions in glycine. **Axes are shown in the shifted φ'-ψ' angles [°]. Energy plots [kcal/mol] of (a) the Lennard-Jones 12-6 potentials of the revised set of steric clashes; (b) all electrostatic interactions; (c)-(f) the individual dipole-dipole interactions of the glycine backbone (see Figure 1A for backbone schematic of the dipoles). Energy parameters were taken from CHARMM22. The light areas show regions of minimum energy.

We thus obtain a revised steric map of glycine, consisting of the steric clashes O_i-1_···O, O_i-1_···N_i+1_, H^α1^···O, H^α2^···O, H^α1^···N_i+1 _and H^α2^···N_i+1_. Using parameters from CHARMM22 [22], we calculate the Lennard-Jones 12-6 potential due to the revised steric clashes (Figure 5A). The minimum-energy region accounts for much of the shape of the observed distribution (Figure 3A).

### Dipole-dipole interactions in glycine

The revised glycine steric map does not explain the diagonal shape of the α, α_L_, β_P_, β_PR _and β_S _regions. In the generic Ramachandran plot, it was found that the diagonal shape of regions could be reproduced using electrostatic dipole-dipole interactions [3] but only when the dipole-dipole interactions were considered individually. The overall electrostatic interaction does not reproduce the observed Ramachandran plot [23]. Here, we use the same approach of treating individual electrostatic dipole-dipole interactions along the glycine backbone.

We calculate the energy map of φ-ψ for the 4 dipole-dipole interactions in the glycine backbone interaction: CO_i-1_···CO, NH···NH_i+1_, CO···NH and CO_i-1_···NH_i+1 _(Figure 5C-F). The electrostatic interactions are calculated with the Lennard-Jones potentials of the steric clashes identified in the section above. We find that the shapes of the different regions of the glycine Ramachandran plot (Figure 3A) are reproduced (Figure 5). The CO···NH interaction produces the diagonal α_L_, α and β_S _region (Figure 5E). The NH···NH_i+1 _interaction also produces a diagonal α_L _and α region (Figure 5D). The α region is symmetric to the α_L _region. The CO_i-1_···CO interaction produces minima corresponding to the β_P _and β_PR _regions (Figure 5C).

In the original glycine steric map (Figure 2A), the region near (φ, ψ) = (-180°, 180°) is forbidden due to a steric clash between O and H. Yet glycine has density in this region in the observed Ramachandran plot (Figure 3A). This can also be seen in the frequency distribution of d(O···H) (Figure 3C), where there is a peak at d(O···H) ~ 2.4 Å. At this peak, the O and H atoms are in contact, as the VDW diameter is 2.5 Å. Thus, in the β_S _region of glycine, the favorable CO···HN dipole-dipole interaction overcomes the steric repulsion of the O and H atoms (Figure 5E).

### The pre-proline Ramachandran plot

Schimmel and Flory argued in 1968 that pre-proline – amino acids preceding proline – has a particularly restricted Ramchandran plot, compared to the generic Ramachandran plot [17]. This was finally observed in the protein database by MacArthur and Thornton (Figure 1B) [2].

There are three main differences between the pre-proline Ramachandran plot and the generic Ramachandran plot. In the pre-proline Ramachandran plot, there is a large excluded horizontal strip at -40° < ψ < 50°, which restricts α_L _and α regions. The α_L _region is shifted up higher. These two features were reproduced in the Schimmel-Flory calculation [17] and subsequent calculations [13,18]. The third feature is a little leg of density poking out below the β-region (Figure 1B; purple in Figure 2C). Karplus called this the ζ region [10], which is unique to pre-proline.

Previous calculations [2,17,18] did not focus on the individual interactions, and did not account for the ζ region. Here, we identify the exact steric clashes that determine the pre-proline Ramachandran plot. We will then analyse the interactions responsible for the ζ region.

### Steric interactions in the pre-proline backbone

In pre-proline, instead of an interaction with the ^N^H atom in the succeeding generic amino acid, the pre-proline interacts with a CH_2 _group of the succeeding proline (Figure 1B). The CH_2 _group exerts a much larger steric effect on the pre-proline Ramachandran plot. MacArthur and Thornton [2] suggested that the dominant effect is due to the N···C^δ^_i+1 _and C^β^···C^δ^_i+1 _steric clashes. Here we can analyse the efficacy of each clash by analysing the statistical distributions directly.

**Figure 6:**
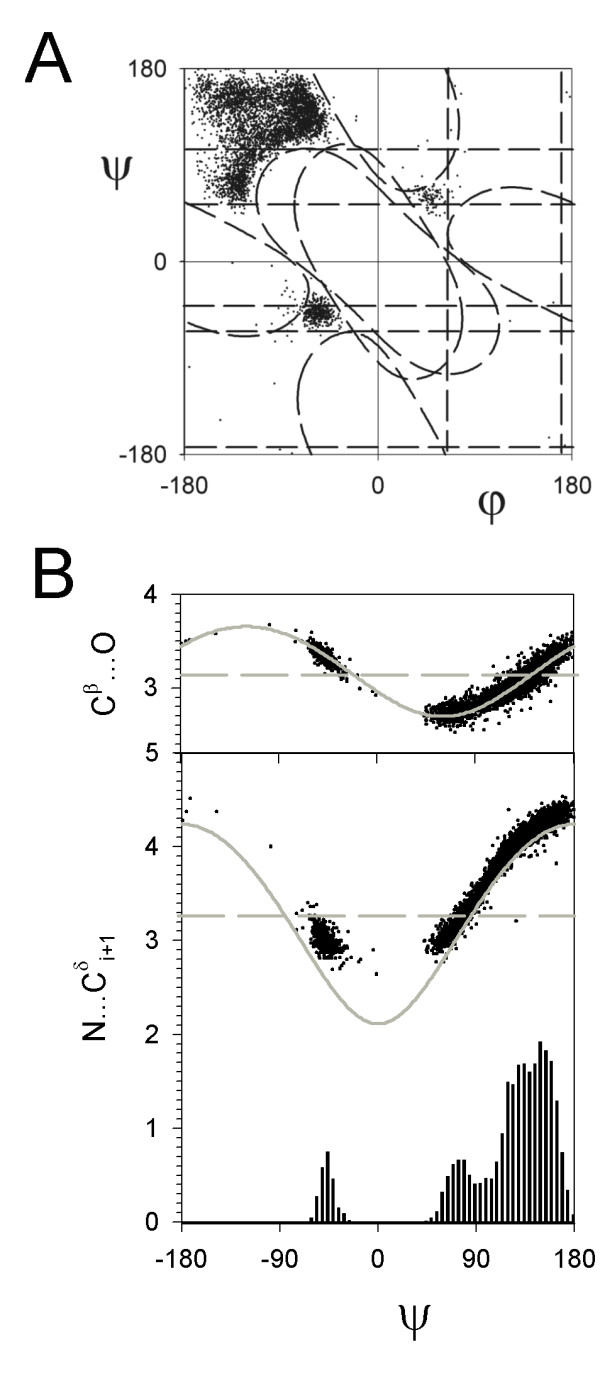
**Pre-proline parameters. **(A) The Ramachandran plot. The dashed lines show the steric clashes that define some of the boundaries of the observed densities (see Figure 2C). (B) The distributions of various inter-atomic interactions as a function of ψ. The dashed lines show the limit of the VDW diameters. The solid grey line gives the model curve calculated with ideal geometry. At the bottom is the frequency distribution of the ψ angle.

We consider the φ-ψ co-dependent interactions that involve the C^δ^, H^δ1 ^and H^δ2 ^atoms of the succeeding proline (Figure 1B). For each interaction, we generate the contour plot in φ-ψ of the VDW diameter distance. By comparing the contour plot to the observed density in the pre-proline Ramachandran plot, we identify the interactions that induce the best match in the boundaries (Figure 6A, the interactions are identified in Figure 2C). We found that the chunk taken out of the bottom-left β-region of the observed density is due to the O_i-1_···C^δ^_i+1 _steric clash. Another restriction on the α_L _and α regions is due to the H···C^δ^_i+1 _steric clash.

We next consider the ψ dependent interactions. In the pre-proline ψ frequency distribution, we found three distinct peaks (bottom Figure 6B). The left-most peak at ψ ~ -50° corresponds to the α region of pre-proline. We focus on the two peaks in the β-region 50° < ψ < 180° The larger peak centred on ψ ~ 150° corresponds to the β_S _region of the generic Ramachandran plot. In the generic Ramachandran plot, this β_S _region is bounded by the C^β^···O and C^β^···N_i+1 _steric clashes. In pre-proline, the smaller peak centred on ψ ~ 70° corresponds to the ζ region and occurs in a region that would be excluded by the C^β^···O steric clash. Instead the smaller peak is bounded from below by the N···C^δ^_i+1 _steric clash. This can be seen by comparing the ψ distribution to the model curve of N···C^δ^_i+1 _vs. ψ (middle of Figure 6B).

**Figure 7:**
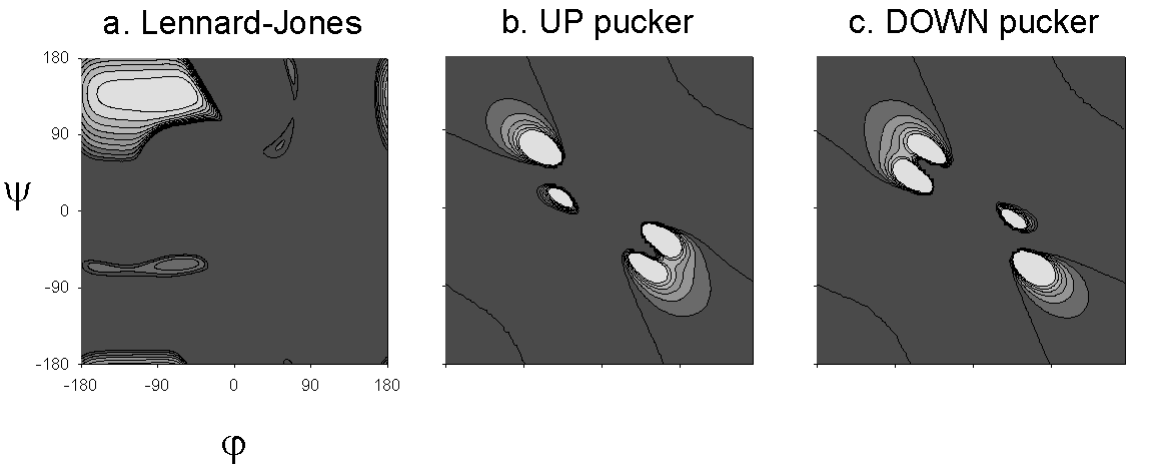
**Energy plots in pre-proline as a function of φ-ψ. **Energy plots [kcal/mol] of (a) the Lennard-Jones 12-6 potentials of the revised set of steric clashes; the CO_i-1_···C^δ^H^δ^_i+1 _dipole-dipole interactions when the succeeding proline ring is in (b) the UP pucker and (c) the DOWN pucker. The light areas show regions of low energy.

Using parameters from CHARMM22, we calculate the Lennard-Jones 12-6 potential due to the revised steric clashes (Figure 7A). Lennard-Jones potentials cannot account for the ζ region.

### Interactions that stabilize the pre-proline ζ region

As the ζ region (purple in Figure 2B) brings the C^β^···O interaction into steric conflict, there must be a compensating interaction that stabilizes the ζ region. What is this interaction? To understand this interaction, we consider an analogy with the γ region in the generic Ramachandran plot. In the γ region, a distorted CO_i-1_···HN_i+1 _hydrogen bond is formed, which brings the H_i+1 _atom into contact with the O_i-1 _atom. Similarly, in the ζ region of pre-proline, the O_i-1 _atom of pre-proline is in contact with the H^δ1 ^and H^δ2 ^atoms (see Figure 4B; Table 1), suggesting that the CO_i-1 _group interacts with the C^δ^H^δ^_i+1 _group of the succeeding proline.

Can the C^δ ^H^δ^_i+1 _group interact with CO_i-1_? Such an interaction would fall under the class of the CH···O weak hydrogen bond, a well-documented interaction in proteins [24]. Studies of the CH···O weak hydrogen bond use a distance criteria of d(H···O) < 2.8 Å [25-27]. There is little angular dependence found in the CH···O bond around the H atom where an angle criteria of ∠OHX > 90° is often used. This is much more permissive than the geometry of the canonical hydrogen bond. In Table 1, we list the hydrogen bond parameters of the CO_i-1_···C^δ^H^δ^_i+1 _interaction in the ζ region. As proline can take on two different major conformations, the UP and DOWN pucker, measurements of the geometry of the CO_i-1_···C^δ^H^δ^_i+1 _interaction must also be divided in terms of the UP and DOWN pucker. The observed geometry of the CO_i-1_···C^δ^H^δ^_i+1 _geometry satisfies the geometric criteria of the weak hydrogen bond (Table 1).

As the CO_i-1_···C^δ^H^δ^_i+1 _weak hydrogen bond is a close contact, we need to model the interaction in order to understand its dependence on the φ-ψ angles. For the modelling, we consider strategies that have been used for the analogous CO_i-1_···HN_i+1 _hydrogen bond. The CO_i-1_···HN_i+1 _hydrogen bond has been modelled in quantum-mechanical studies where the γ region was found to be the minimum energy conformation in vacuum [12]. A simpler approach, which modelled the hydrogen bond with electrostatic dipole-dipole interactions, also find a minimum in the γ region [23].

Here, we model the CO_i-1_···C^δ^H^δ^_i+1 _weak hydrogen bond as an electrostatic dipole-dipole interaction (see Methods). How do we model the C^δ^H^δ^_i+1 _group as an electrostatic dipole? Bhattacharyya and Chakrabarti [28] found that, of the CH groups in proline, the C^δ^H^δ ^group forms the most CH···O hydrogen bonds. The C^δ ^atom sits next to the electron-withdrawing N atom and thus, is more acidic than the other C atoms. Consequently, we place a small negative partial charge on the C^δ ^atom. In our model, we find an energy minimum in the ζ region for both the UP pucker (Figure 7B) and the DOWN pucker (Figure 7C). We conclude that the CO_i-1_···C^δ^_i+1_H^δ1^_i+1 _weak hydrogen bond stabilizes the ζ region in pre-proline.

## Conclusion

We have identified the interactions that determine the high-resolution Ramachandran plots of glycine and pre-proline.

For glycine, the Ramachandran plot of the glycine backbone modeled by standard force-fields fails to reproduce the observed Ramachandran plot [16]. Instead the modeled Ramachandran plot resembles the original steric map of glycine [1]. The failure of these calculations arises from the inadequate treatment of the H^α ^atoms. We have identified a revised set of steric interactions that can reproduce the observed glycine Ramachandran plot. These are O_i-1_···O, O_i-1_···N_i+1_, H^α1^···O, H^α2^···O, H^α1^···N_i+1 _and H^α2^···N_i+1 _(Figure 2B). These steric interactions constrain either the N_i+1 _or O atom to be sandwiched between the two H^α ^atoms, which clusters glycine to ψ = 180° and ψ = 0°. The five clustered regions can be traced to electrostatic dipole-dipole interactions: the CO···NH interaction induces diagonal α_L_, α and β_S _regions; and the CO_i-1_···CO interaction induces the diagonal β_P _and β_PR _regions.

Previous calculations of the pre-proline Ramachandran reproduced most of the observed pre-proline Ramachandran plot with the notable exception of the ζ region. Previous studies did not identify the specific steric interactions involved in defining the pre-proline Ramachandran plot. Here, we have identified them: N···C^δ^_i+1_, O_i-1_···C^δ^_i+1 _and H···C^δ^_i+1 _(Figure 2C). We have also identified the physical mechanism that stabilizes the ζ region (purple in Figure 2C). It is the CO_i-1_···C^δ^H^δ^_i+1 _weak hydrogen bond, which is directly analogous to the CO_i-1_···NH_i+1 _hydrogen bond that stabilizes γ-turns in the generic amino acid.

Combined with the analysis of the generic Ramachandran plot [3] and the proline Ramachandran plot [13,14], we have identified the interactions that define the high-resolution Ramachandran plots of all 20 amino acids. Although our analysis uses simple modeling techniques, the interactions identified here suggest concrete ways to resolve the inadequacies in current force-fields.

## Methods

### VDW radii

In the steric clash analysis, we used the VDW radii given by the Richardson lab [29]: H^α ^= 1.17Å, H = 1.00Å, C = 1.65Å, C^α ^= C^β ^= 1.75Å, O = 1.40Å and N = 1.55Å. From the database, we extracted 7277 glycine and 4336 pre-proline residues.

### Local conformations of the φ-ψ map

To calculate the model curves of the inter-atomic distances as a function of the φ-ψ angles, we modeled the glycine and pre-proline protein fragments shown in Figure 1. Covalent bond lengths and angles were fixed to CHARMM22 values [22]. Only the φ-ψ angles vary. The φ-ψ angles of the central residue were incremented in 5° steps and the corresponding distance parameters and energies of the inter-atomic interactions were calculated. We used 2 types of interactions, partial charge electrostatics, E_elec _= 331·(q_1_·q_2_) kcal·mol^-1^, and Lennard-Jones 12-6 potentials, E_LJ _= ε (σ/d)^12 ^– 2 (σ/d)^6^) kcal·mol^-1^, where the parameters were taken from CHARMM22 [22]. There are no parameters in CHARMM22 for the H^δ ^and C^δ ^atoms. As such, we have assigned a partial charge of -0.20 to C^δ ^and 0.10 to H^δ1 ^and H^δ2^. These are not based on any detailed arguments but are merely used to estimate the effect that such charges would have.

## Authors' contributions

Both authors conceived the study. BKH carried out the data analysis and modelling, and drafted the manuscript. RB provided guidance and mentorship.

## Figures and Tables

**Table 1:** Parameters of the CO···HX hydrogen bond

	**n**	**φ**	**ψ**	**O···H**	∠**COH**	∠**OHX**	∠_dih_**COHX**
	γ *region of the generic amino acid*						
CO···HN	518	-85(3)°	81(11)°	2.39(0.24) Å	107(5)°	123(10)°	178(11)°
							
	ζ *region of pre-proline: UP PUCKER*						
CO···H^δ1^C^δ^	105	-129(6)°	80(6)°	3.64(0.27) Å	79(8)°	75(10)°	25(9)°
CO···H^δ2^C^δ^				2.59(0.22) Å	99(12)°	144(14)°	-108(18)°
							
	ζ *region of pre-proline: DOWN PUCKER*						
CO···H^δ1^C^δ^	406	-129(6)°	74(9)°	3.16(0.38) Å	95(32)°	101(20)°	22(11)°
CO···H^δ2^C^δ^				2.98(0.36) Å	71(10)°	113(22)°	-118(14)°
